# Bio-Fenton reaction involved in the cleavage of the ethoxylate chain of nonionic surfactants by dihydrolipoamide dehydrogenase from *Pseudomonas nitroreducens* TX1

**DOI:** 10.1038/s41598-019-43266-8

**Published:** 2019-05-02

**Authors:** Kuo-Chan Hung, Ngoc Tuan Nguyen, Yu-Ling Sun, Shir-Ly Huang

**Affiliations:** 1 Department of Life Sciences, National Central University, Jhongli, Taiwan; 2 Institute of Microbiology and Immunology, National Yang-Ming University, Taipei, Taiwan; 3 Institute of Environmental Engineering, National Central University, Jhongli, Taiwan; 4 Present Address: Faculty of Applied Sciences, Ton Duc Thang University, Ho Chi Minh City, Vietnam

**Keywords:** Applied microbiology, Soil microbiology

## Abstract

Bacteria in the environment play a major role in the degradation of widely used man-made recalcitrant organic compounds. *Pseudomonas nitroreducens* TX1 is of special interest because of its high efficiency to remove nonionic ethoxylated surfactants. In this study, a novel approach was demonstrated by a bacterial enzyme involved in the formation of radicals to attack ethoxylated surfactants. The dihydrolipoamide dehydrogenase was purified from the crude extract of strain TX1 by using octylphenol polyethoxylate (OPEO_n_) as substrate. The extent of removal of OPEOs during the degradation process was conducted by purified recombinant enzyme from *E. coli* BL21 (DE3) in the presence of the excess of metal mixtures (Mn^2+^, Mg^2+^, Zn^2+^, and Cu^2+^). The metabolites and the degradation rates were analyzed and determined by liquid chromatography-mass spectrometry. The enzyme was demonstrated to form Fenton reagent in the presence of an excess of metals. Under this *in vitro* condition, it was shown to be able to shorten the ethoxylate chains of OPEO_n_. After 2 hours of reaction, the products obtained from the degradation experiment revealed a prominent ion peak at *m*/*z* = 493.3, namely the ethoxylate chain unit is 6 (OPEO_6_) compared to OPEO_9_ (*m/z* = 625.3), the main undegraded surfactant in the no enzyme control. It revealed that the concentration of OPEO_15_ and OPEO_9_ decreased by 90% and 40% after 4 hours, respectively. The disappearance rates for the OPEO_n_ homologs correlated to the length of the exothylate chains, suggesting it is not a specific enzymatic reaction which cleaves one unit by unit from the end of the ethoxylate chain. The results indicate the diverse and novel strategy by bacteria to catabolize organic compounds by using existing housekeeping enzyme(s).

## Introduction

Octylphenol polyethoxylates (OPEO_n_, commercial name Triton X-100) is a common nonionic surfactant that is used in industrial and household products^1,2^. Its structures is made of hydrophilic polyethoxylate chain (on average it has 9.5 ethylene oxide units) bound to a hydrophobic moiety (octylphenol)^3^. In the environment, OPEO_n_ can be degraded into shorter exothylate chain to form octylphenol or octylphenol mono- to tri-ethoxylates^4^. These OPEO_n_ metabolites that are known more estrogenic-like than their parent compounds can mimic the natural hormones and thus act as endocrine disruptors in wildlife^5,6^.

Much attention has been paid to the fate and degradability of OPEO_n_ in the environment^7^. The biodegradation of OPEO_n_ has been studied using both pure and mixed cultures that grow solely on OPEO_n_. Several bacterial strains have been reported to degrade the ethoxylate (EO) chain of OPEO_n_ ^1,7–11^. The oxidation models have been proposed based on biodegradation intermediate metabolites^12–15^. In our previous studies, *P. nitroreducens* TX1 which revealed an ability to grow on 0.05–20% OPEO_n_ as a sole carbon source^1,8–11,16^. The formation of carboxylated octyl-moiety from the catabolism of octylphenol polyethoxylates by *P. nitroreducens* TX1 was described. The LC–MS analysis shows that the ethoxylate chain was shortened and the estrogen-like intermediates were produced. A library containing 30,000 Tn5-insertion mutants of the wild-type strain TX1 was also constructed and screened for OPEO_n_ utilization. The result revealed the role of the glyoxylate cycle in OPEO_n_ degradation^15^. However, the evidence for the shortening of ethoxylate units is still lacking. In this study, we investigated the enzyme activities involved in OPEO_n_ degradation in strain TX1 and a mechanism involved in the degradation of polyethoxylate chains was reported. The extent of removal for residual OPEOs during the degradation process by a pure recombinant enzyme was also investigated.

## Materials and Methods

### Chemicals

Triton X-100 was purchased from Merck Chemical Co. (Darmstadt, Germany). The average number of ethoxylate (EO) units for Triton X-100 is 9.5 according to the manufacturer’s information, which corresponds to an average molecular weight of ca. 625. All the reagents were purchased from the Merck Chemical Co. (Darmstadt, Germany) at a purity of 98–99.5%.

### Bacterial strains and culture conditions

*P. nitroreducens* TX1 was isolated from a rice field drainage in Taiwan^10^. *E. coli* strains DH5alpha, BL21(DE3) (Invitrogen, Carlsbad, CA) were used in cloning and expression. *E. coli* HB101 (pRK2013) was used as a helper in triparental mating experiments^17^. Luria-Bertani (LB) and mineral salts basal (MSB) media were described in the previous studies^8,11^. For the large-scale cultivation of strain TX1, a stirred bioreactor was used. The inoculum (10% of the working volume) was transferred from the flask of the one-day old culture to the bioreactor, which contained 3 L of the desired medium. Cultivations were conducted in a 5 L stirred bioreactor (BTF-A5L, Bio-Top Inc, Taiwan) at 200 rpm and with aeration of 0.3 vvm (volume air/volume liquid/min) in the MSB medium containing 0.5% OPEO_n_. The fermentation broth was harvested at late log phase for the enzyme purification.

### Preparation of crude cell extract and purification of enzyme involved in OPEO_n_ degradation from strain TX1

Strain TX1 was cultivated in 20 L of MSB-0.5% OPEO_n_ medium for the preparation of crude cell extract. The cells were collected by centrifugation (11,000 *g*, 10 min, 4 °C), washed with 40 mM Na_2_HPO_4_/KH_2_PO_4_ buffer (pH 7.0) and suspended in 800 mL of the same buffer. The cell crude extract was prepared as in our previous study^18,19^ and then treated with 0.3% protamine sulfate. The supernatants were collected at 30,000 *g* for 30 minutes for enzyme purification. All purification procedures were performed at 4 °C in Na_2_HPO_4_/KH_2_PO_4_ buffer (pH 7.0) unless otherwise stated. Enzymes were loaded onto a DEAE-Sepharose XK 26 column (155 mL). The column was preequilibrated with buffer and enzymes with active fractions were eluted with 1 M KCl. The active fractions were collected and were fractionated with ammonium sulfate. The precipitate obtained with 0 to 60% saturation of ammonium sulfate was collected and applied to a phenyl Superpose 6 fast flow column (60 mL). The active fractions were eluted with a linear gradient of 1 to 0 M (NH_4_)_2_SO_4_. The concentrated enzyme solution was loaded onto a Sephacryl S-200 (176 mL) for the next purification step. The active fractions were eluted with pH 6.9 buffer. Finally, the active fractions after gel filtration were loaded onto a Mono P HR 5/20 chromatography column (4 mL). The column was preequilibrated with 25 mM diethanolamine-HCl (pH 9.5) and was eluted with 100 mL polybuffer 96 (Pharmacia Fine Chemicals) and titrated with HCl to pH 6.0.

### Protein quantification, in-gel digestion and protein identification

A 12% SDS-PAGE gel was used for determination of molecular weight of the purified enzyme and enzyme purity. After separation in SDS-PAGE gels, the proteins were visualized by staining using mass compatible Comassie blue. Excised gel pieces were washed with deionized water twice, then destained with 200 μL ammonium bicarbonate (ABC, NH_4_HCO_3_)/50% v/v acetonitrile (ACN, CH_3_CN) for 15 min and dehydrated by incubation with 100 μL 100% ACN for 5 min. The process was repeated until gels were destained completely. The gel pieces were further dried by vacuum concentration for 10 min. Prior to the tryptic digestion, the sample was diluted with 25 mM ABC to give a final urea concentration of less than 0.6 M. Trypsin was added with weight ratio of 20 (protein): 1 (trypsin) and the sample was incubated in a waterbath at 37 °C for 12–16 h. The reaction was stopped with 10 μL 0.1% formic acid. The digested peptides were extracted by ultrasonication for 5 min and stopped for 5 min (repeat three times) and the peptides were kept in the supernatant in the new tube. The sonication procedure was repeated by adding 0.1% formic acid/50% ACN. The peptides were concentrated by vacuum concentration. The resulting peptide mixture was then subjected to the CapLC system (Waters, Milford, MA, USA) utilizing a capillary column (75 µm i.d., 10 cm in length) with a linear gradient from 5 to 50% ACN containing 0.1% formic acid over 46 min. The separated peptides were on-line analyzed under positive survey scan mode on a nano-ESI-Q-TOF instrument (Micromass, Manchester, UK). The scan range was from *m*/*z* 400 to1600 for MS and *m*/*z* 50 to 2000 for MS/MS. The raw data were acquired and processed using MassLynx V 4.1 software (Micromass) and were converted to PKL files by the ProteinLynx 2.2.5 software. The PKL files were analyzed using the MASCOT search engine (http://www.matrixscience.com). The proteins with scores above the significant threshold (*P* < 0.05) are shown as identified proteins.

### Enzyme assay and hydrogen peroxide assay

Enzyme activity was assayed by oxygen uptake rate in a 1.5 mL reaction mixture containing 50 mM KH_2_PO_4_/Na_2_HPO_4_ (pH 8.0), 10 μM MgSO_4_, 5 μM Co(NO_3_)_2_, 100 μM FeSO_4_, 200 μM ZnSO_4_, 10 μM CuSO_4_, 60 μM MnSO_4_, 40 μM Na_2_EDTA, 0.05% OPEO_n_, 144 µg enzyme and 0.5 mM NADH at 30 °C by an oxygen monitor (Biological Oxygen Monitor, Yellow springs co. Ohio, USA). To estimate the kinetic parameters of the enzyme, the pure enzyme activity was measured in the forward reactions according to the literature^20^. Protein concentrations were determined using the Bradford protein assay with bovine serum albumin as the standard^21^. Specific enzyme activity is reported as nmole/min/mg. The hydrogen peroxide assay was based on the detection of H_2_O_2_ using the amplex red fluorescent dye^22^. In the presence of horseradish peroxidase, the amplex red reagent reacts with H_2_O_2_ with a 1:1 stoichiometry producing highly fluorescent resorufin. 1.5 µg pure enzyme were added into 0.25 mL reaction mixture containing 50 mM KH_2_PO_4_/Na_2_HPO_4_ (pH 8.0), 0.05 mM NADH, 0.2 mM ZnCl_2_ at 30 °C for 1 h and then horseradish peroxidase (0.2 U/ml) and amplex red reagent (1 µM) were added. Production of resorufin was followed by an increasing absorbance at 571 nm. Concentrations of H_2_O_2_ were calculated by comparing absorbance of samples to a series of H_2_O_2_ standards (0.2~1 µM) treated with the amplex red mixture.

### Construction of bacterial strains

To inactivate the lipoamide dehydrogenase (*lpd*) gene in wild-type TX1, a gene fragment containing about 400 bp of the internal region of *lpd* gene (accession number WP_017518066.1) was amplified and cloned into pK18mobsacB plasmid. The primers used were F_lpd: 5′-GCGAATTCGAAGACCCTGACCAAGCAAG (EcoRI, underlined); R_lpd: 5′-GCAAGCTTATTTCCGGGTGGGTGTAGAT (HindIII, underlined). The gene fragments were cloned at the EcoRI/HindIII site into the pK18mobsacB plasmid. The resulting plasmid was named pKlpd. The lpd-internal mutation of TX1 was created by triparental mating between strains TX1, *E. coli* DH5α (pKlpd) and *E. coli* HB101 (pRK2013) as previously described^23^. Transconjugants were screened on LB containing both ampicillin and kanamycin, and finally confirmed by PCR.

To clone the *lpd* gene into the pET28a vector, a method termed “sticky-end PCR”, which generates PCR product bearing cohesive ends compatible with any intended restriction sites, was used^24^. Briefly, two pairs of PCR primers differing only in the 5′ ends were designed to amplify *lpd*. PCR was performed in two separate tubes using primer set 1 (F1: 5′-CATGAGCCAGAAATTCGACGTG-3′ and R1: 5′-GGCGCTTCTTGCGGTTGGC-3′) and set 2 (F2: 5′-AATTCATGAGCCAGAAATTCGACGTG-3′ and R2: 5′-TCGAGGCGCTTCTTGCGGTTGGC-3′), respectively. The differences in the 5′ termini of the primers matched the overhangs generated by the EcoRI and XhoI cleavage are underlined in parentheses. The two PCR products were then mixed, followed by denaturation and annealing, which resulted in half of the mixed products bearing cohesive ends. The mixed DNA fragment was ligated with an EcoRI/XhoI-digested pET28a plasmid. The resulting plasmid was named pElpd and then transformed into *E. coli* BL21 (DE3).

### Purification of Lpd in recombinant *E*. *coli*

The cell crude extract from *E. coli* BL21 was filtered through a 0.22 µm filter and loaded onto HisTrap affinity column (5 mL; GE Healthcare). The unabsorbed and loosely bound proteins were eluted from the column by washing with 5 column volumes of 30 mM imidazole in 20 mM KH_2_PO_4_/Na_2_HPO_4_ buffer (pH 7.0). His-tag Lpd protein was eluted with 500 mM imidazole in the same buffer. All the fractions containing Lpd activity were combined. To further purify the enzyme, gel filtration was performed with a Superose 6 column that was preequilibrated with 50 mM KH_2_PO_4_/Na_2_HPO_4_ at pH 7.0. The proteins were eluted with the same buffer and all the active fractions were combined. All procedures were done at 4 °C.

### Identification of degradation products from OPEO_n_

To carry out the analysis of degradation products, each 4 mL enzyme reaction (enzyme activity was performed in a reaction mixture containing 50 mM KH_2_PO_4_/Na_2_HPO_4_ (pH 8.0), 10 μM MgSO_4_, 5 μM Co(NO_3_)_2_, 100 μM FeSO_4_, 200 μM ZnSO_4_, 10 μM CuSO_4_, 60 μM MnSO_4_, 40 μM Na_2_EDTA, 0.05% OPEO_n_, 96 µg/mL pure Lpd enzyme and 0.5 mM NADH at 30 °C) at its particular time point was mixed vigorously with 10 mL of 72.4% of MgSO_4_ and 0.2 mL of 2.5 M H_2_SO_4_ followed by extraction with 25 mL of chloroform (CHCl_3_) three times^14^. A portion of the organic phase was collected and dried immediately using a rotary evaporator. Acetonitrile (2 mL) was then added to dissolve the dry residue for liquid chromatography– mass spectrometry (LC–MS) analysis. OPEO_n_ and its degradation products were analyzed by a high-performance liquid chromatography (HPLC) system (Waters Alliance 2690, Milford, MA, USA) equipped with an electrospray ionization– mass spectrometer (Platform LC; Micromass, Manchester, UK). The injection volume was 20 µL and the flow rate was set at 0.5 mL/min. A 5 µm C_18_ column (Waters µBondapak, 3.9 × 150 mm) was used for the separation. The OPEO_n_ was analyzed in the positive mode. In the positive mode, a mobile phase containing 0.1% aqueous formic acid and acetonitrile (3:7) was applied to analyze OPEO_n_ and the products. The potentials of the electrospray ionization source were set at 3.5 V for the capillary voltage and 50 V for the cone voltage. The source temperature was 100 °C and the flow rate of nitrogen gas was controlled at 300 L h^−1^.

## Results

### Existence of metabolic enzymes related to OPEO_n_ degradation in strain TX1

*P. nitroreducens* TX1 is of special interest because of its capability to use 0.05–20% OPEO_n_ as a sole carbon source^1,8–11,16^. In this study, oxygen consumption activity was used to evaluate the involvement of O_2_ in the degradation of OPEO_n_ in a whole cell system. The result revealed that OPEO_n_-dependent oxygen consumption activity induced in TX1 cells prepared from MSB containing 0.5% OPEO_n_ (33.8 nmole/min) was 3.7 fold higher than that grown on 0.5% succinate (Table 1). These finding suggested the presence of metabolic enzymes in the TX1 cell which are related to the growth on OPEO_n_ as sole carbon source. Using oxygen consumption activity as an enzyme assay, we attempted to purify the metabolic enzymes involved.

**Table 1:** Oxygen uptake in *Pseudomonas nitroreducens* TX1 grown on different concentrations of OPEO_n_ or succinate.

Carbon source^a^ (%, v/v)		Oxygen uptake^b^ (nmole/min)	Relative rate (fold)
Succinate	OPEO_n_		
0.5	0	9.1 ± 2.6	1
0	0.005	15.8 ± 3.5	1.7
0	0.05	13.1 ± 2.6	1.4
0	0.5	33.8 ± 1.7	3.7
0	2.5	29.6 ± 1.7	3.3
0	5	8.8 ± 1.3	1

^a^*Pseudomonas nitroreducens* TX1 was cultivated in minimal salts basal medium (MSB) with different concentration of OPEO_n_ or succinate at 30 °C and harvested at log phase.

^b^The oxygen uptake is determined in a TX1 cell suspension (5 mL, OD_600 nm_ = 0.3) containing 0.05% OPEO_n_. The reaction rate was analyzed in MSB at 30 °C for 1 min.

### Purification of enzyme involved in OPEO_n_ degradation from strain TX1

**Figure 1:**
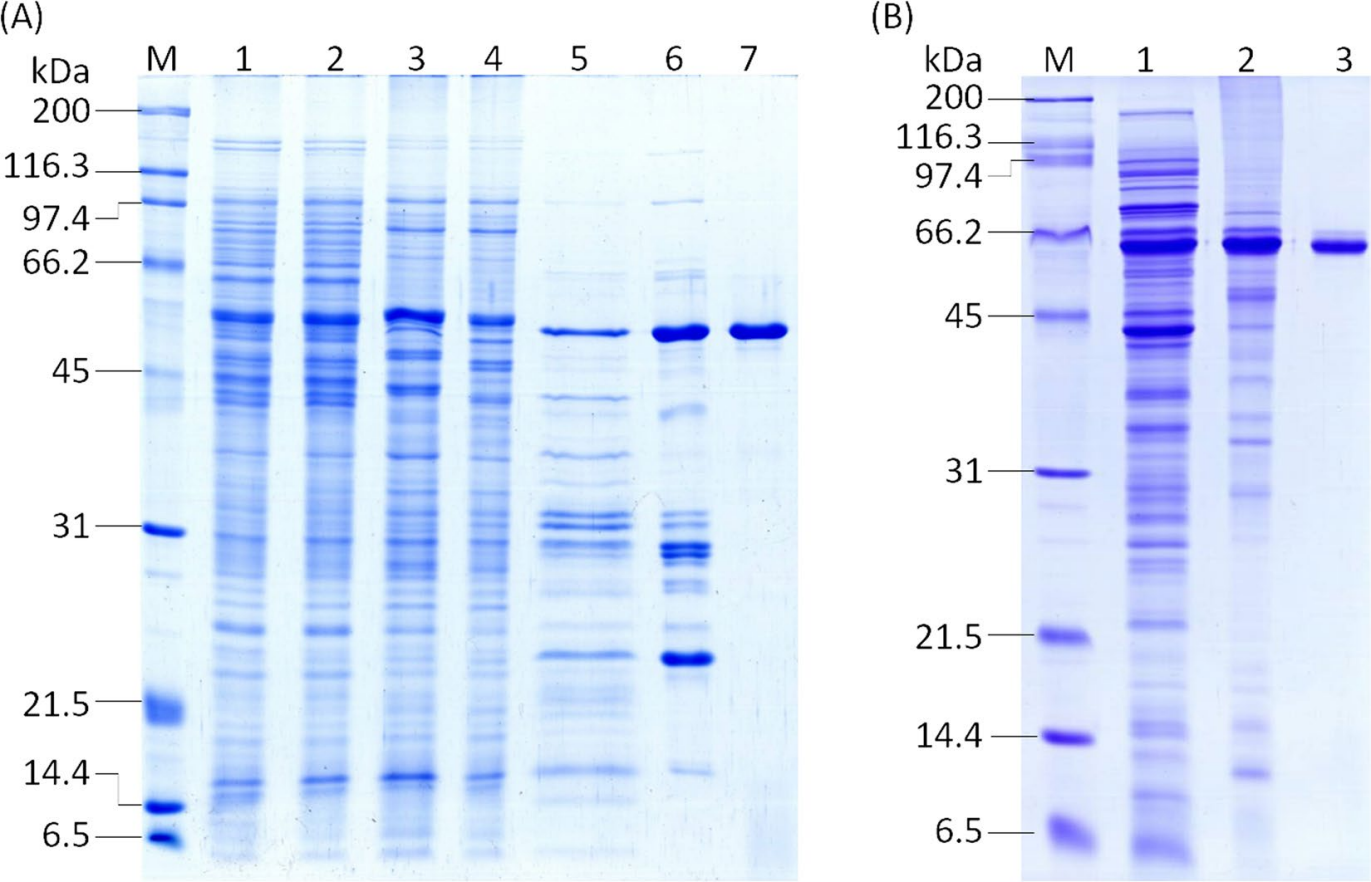
(**A**) 12% SDS-PAGE of the purification steps from *P. nitroreducens* TX1. Lane 1: Crude cell extract of strain TX1; lane 2: protamine sulfate treated; lane 3: DEAE-Sepharose; lane 4: 25–60% ammonium sulfate; lane 5: Phenyl-Sepharose; lane 6: Sephacryl S-200; lane 7: Mono P HR 5/20; lane M: molecular mass markers. (**B**) 12% SDS-PAGE of the purification steps from recombinant *E. coli* BL21 (DE3). Lane 1: Crude cell extract of recombinant cells; lane 2: His-trap; lane 3: Superose 6. The protein sample contains 5 μg in each well. The gel was stained by Coomassie Brilliant blue.

The cell-free extract of strain TX1 was prepared from 20 L culture. The purification steps for the enzyme are summarized in Table 2. Enzyme activity was assayed by oxygen consumption as described in “Materials and Methods”. The enzyme was purified 18-fold with a 0.4% yield that appeared to be highly purified on an SDS-PAGE gel (Fig. 1). The specific activity of the purified enzyme was 41.3 nmol/min/mg. On SDS-PAGE, the molecular mass of a monomeric protein was estimated to be 52 kDa. By gel filtration, the active fraction was eluted at a retention time corresponding to approximately 98 kDa. The finding suggested that the enzyme exists as a dimer in the native state. The optimum temperature and pH were found to be 30 °C and 8.0, respectively. The effects of various metal ions on purified enzyme activity were examined (Table 3). Some metal ions (Mn^2+^, Mg^2+^, Zn^2+^, and Cu^2+^) enhanced enzyme activity. Excess of EDTA was checked and revealed a similar activity as the “no metals” control.

**Table 2:** Purification of OPEO_n_ - oxygen consuming enzyme from *P. nitroreducens* TX1.

Purification step	Volume (ml)	Total protein (mg)	Total activity^a^ (nmol/min)	Specific activity (nmole/min/mg)	Recovery (%)	Purification (fold)
Crude extract	803	4818	11202	2.3	100	1
Protamine sulfate	875	4210	11725	2.8	105	1.2
DEAE-Sepharose	2340	1614.6	7956	4.9	71	2.1
25–60% (NH_4_)_2_SO_4_	27	638.6	3510	5.5	31.3	2.4
Phenyl-Sepharose	171	70.11	940.5	13.4	8.4	5.8
Sephacryl S-200	12.6	12.85	189	14.7	1.7	6.4
Mono P	3.2	1.14	47	41.3	0.4	18.0

^a^Enzyme activity was assayed by oxygen consumption rate in a 1.5 mL reaction mixture containing 50 mM KH_2_PO_4_/Na_2_HPO_4_ (pH 8.0), 10 μM MgSO_4_, 5 μM Co(NO_3_)_2_, 100 μM FeSO_4_, 200 μM ZnSO_4_, 10 μM CuSO_4_, 60 μM MnSO_4_, 40 μM Na_2_EDTA, 0.05% OPEO_n_, 144 µg DLDH enzyme and 0.5 mM NADH at 30 °C.

**Table 3:** Metal effect on purified enzyme analyzed by oxygen consumption activity.

Trace metals	Activity^b^	
	nmole/min	%
No metals	0.89	100
Trace metals solution^a^	2.47 ± 0.14	278 ± 16
FeSO_4_	—^c^	—^c^
MnSO_4_	2.88 ± 0.06	324 ± 7
MgSO_4_	2.10 ± 1.00	236 ± 112
ZnSO_4_	1.59 ± 0.23	179 ± 26
CuSO_4_	1.67 ± 0.01	188 ± 1
Co(NO_3_)_2_	0.98 ± 0.34	110 ± 42
Na_2_EDTA	1.00 ± 0.09	112 ± 10

^a^The trace metals solution is composed of 10 μM MgSO_4_, 100 μM FeSO_4_, 60 μM MnSO_4_, 200 μM ZnSO_4_, 10 μM CuSO_4_, 5 μM Co(NO_3_)_2_, 40 μM Na_2_EDTA.

^b^Enzyme activity was measured by oxygen consumption rate in a 1.5 ml reaction mixture containing 50 mM KH_2_PO_4_/Na_2_HPO_4_ (pH 8.0), 1 mM OPEO_n_, 0.5 mM NADPH, 34 μg purified enzyme and additional metals at 37 °C. No metals in the reaction mixture is used as 100%. All data are in triplicate.

^c^The basal oxygen consumption of the enzyme activity assay in the presence of FeSO_4_ is too fast to serve as initial O_2_ consumption rate for the activity monitoring.

### Analysis of degradation products from pure enzyme

**Figure 2:**
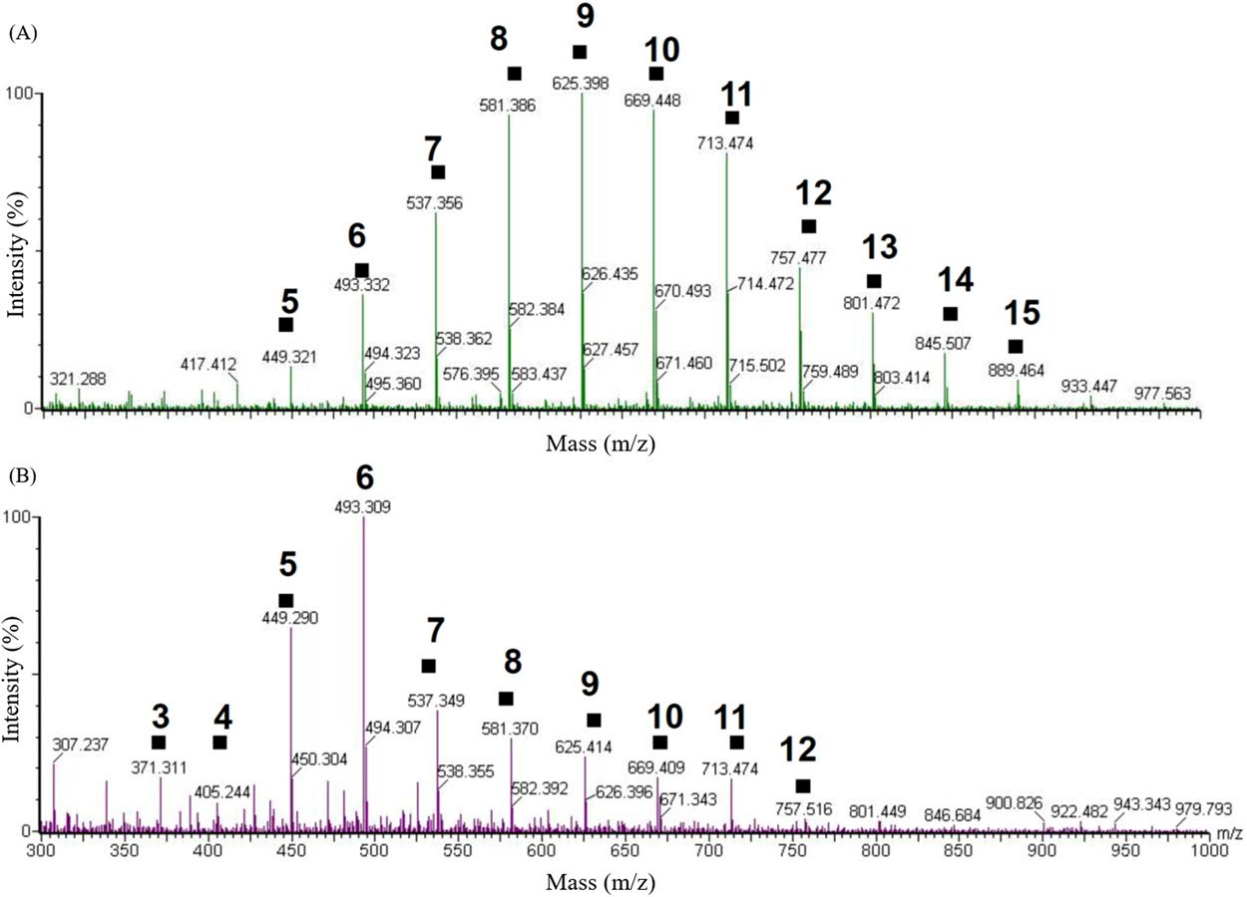
Mass spectra of OPEO_n_ degradation by the purified enzyme in the present of metals and NADH. (**A**) At zero time, the mass spectrum of reaction shows a characteristic pattern of the OPEO_n_ parental ion pattern. The most abundant ethoxymer is OPEO_9_ (m/z = 625.3). (**B**) After 2 hours reaction, the profile shifts to lower EO chain products with OPEO_6_ (m/z = 493.3) as most abundant ethoxymer. The reaction mixture (4 mL) contains 50 mM KH_2_PO_4_/Na_2_HPO_4_ (pH 8.0), 10 μM MgSO_4_, 5 μM Co(NO_3_)_2_, 100 μM FeSO_4_, 200 μM ZnSO_4_, 10 μM CuSO_4_, 60 μM MnSO_4_, 40 μM Na_2_EDTA, 0.05% OPEO_n_, 96 µg/mL pure Lpd enzyme and 0.5 mM NADH at 30 °C.

The products of OPEO_n_ degraded by the enzyme in the presence of metals and an excess of NADH were analyzed by HPLC-MS. Figure 2 shows mass spectra of the OPEO_n_ at zero time (**A**) and that after the incubation for 2 h (**B**). In both mass spectra, a series of intense ion peaks (■) of OPEO_n_ molecules with a sodium cation attached [R-(CH_2_CH_2_O)_n_-H + Na^+^] are clearly observed. However, compared with the mass spectrum of the original sample, the ion peak distribution of mass spectra obviously shifts to lower mass with the elapse of the incubation time. After 2 hours of reaction, the products obtained from the degradation experiment revealed a prominent ion peak at *m*/*z* = 493.3, corresponding to OPEO_6_. When comparing OPEO_n_ without the presence of the enzyme, the products were mainly OPEO_9_ (m/z = 625.3), the dominant form in the original substrate. Mass spectra of the reaction showed a characteristic pattern due to loss of individual EO [CH_2_-CH_2_-OH] units of m/z = 44.0 from the parental OPEO_n_. The shortest detectable compound in our condition is OPEO_3_ (*m*/*z* = 371.3). However, the other product with smaller molecular mass is not detected in this study. The reasons might be that small EO units are (1) lost in extraction steps; (2) co-eluted with initial solvent peak in LC. The degradation products detected in this study indicate that the degradation mechanism for OPEO_n_ by a pure enzyme from strain TX1 involves the shortening of ethoxylate units in the polyethoxylate chains.

### Protein identification

The identity of the protein band was determined by partial amino acid sequence via ESI-MS/MS. The major protein in the 52-kDa band matched to dihydrolipoamide dehydrogenase from *P. nitroreducens* TX1 (accession number WP_017518066), which had the highest Mowse score and the largest number of peptide matches (31 peptide matches). These peptide matches cover 47% of the protein and are spread throughout the protein (Supplemental Fig. S1). The calculated molecular weight of Lpd (50 kDa) is in close agreement with that observed in our SDS-PAGE analysis. The absorption spectrum of the purified enzyme shows maximal absorbance at 375 and 458 nm. As the enzyme had high homology with Lpds, the Lpd activity was confirmed according to the literature^20^. When the concentrations of NAD^+^ and dihydrolipoamide were varied, the initial velocities represented a series of parallel lines, suggestive of a ping-pong reaction mechanism^25^. The result revealed the kinetic parameters of Lpd (*K*_M, NAD_^+^ = 0.52 mM; *V*_max_ = 0.09 µmole/min) (Fig. S2). The specific activity of Lpd in this experiment was 108.2 µmole/min/mg which was higher than that from *P*. *fluorescens* (35.7 µmole/min/mg)^26^ and *P*. *putida* (68 µmole/min/mg)^27^.

### Analysis of the genome fragment containing the *lpd* gene and insertion mutagenesis

**Figure 3:**
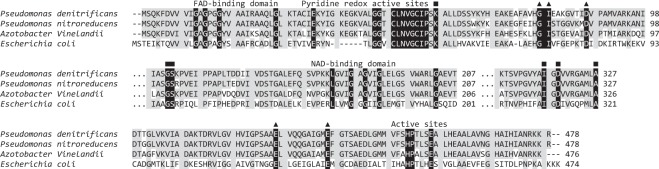
The amino acid sequence alignment of dihydrolipoamide dehydrogenase from *Pseudomonas nitroreducens* TX1 with these from *Pseudomonas denitrificans* (GenBank accession number WP_015477517.1), *Azotobacter Vinelandii* (3LAD) and *Escherichia coli* (P00391). Identical amino acid residues are highlighted in gray. ▲, the residues involved in the dimerization; ■, the residues involved in FAD binding.

According to the draft genome sequence from strain TX1^16^, the *lpd* gene locates in contig099 (accession number NZ_AMZB01000099). A 7.9 kb fragment located in the 57613–65519 position of contig099 was analyzed (Fig. S3). The succinate dehydrogenase, 2-oxoglutarate dehydrogenase, and dihydrolipoamide succinyltransferase genes were located upstream of *lpd*, whereas the malate-CoA ligase gene was located downstream of *lpd*. All genes are involved in the TCA cycle. The *lpd* consists of 1,437 bp (Fig. S4), encoding 478 amino acids. A search of a nucleotide sequence database (http://www.ncbi.nlm.nih.gov/BLAST) was performed and revealed that *lpd* had the highest similarity (94%) to the dihydrolipoamide dehydrogenase gene from *P. denitrificans* (WP_015477517.1). For different genera, the highest similarity (83%) to the dihydrolipoamide dehydrogenase gene from *Azotobacter vinelandii* (3LAD) was observed. The sequence comparison was carried out and revealed that the FAD-binding domain (Gly-11, 13 and 16), the NAD-binding domain (Gly-186, 188, 191 and 202) and the residues involved in the dimerization are completely conserved in Lpd (TX1), as compared to others (Fig. 3). A redox active center containing two cysteine residues (Cys-49 and Cys-54) are directly engaged in thiol-disulfide exchange reactions during catalysis. To further confirm the role of *lpd* gene on the growth of TX1 with OPEO_n_, efforts in inactivate the *lpd* gene were made, but no mutant was found, suggesting that Lpd is essential to cell.

### Characterization of recombinant Lpd

Because of the low yield (0.4%) of purified protein from wild type TX1 and the long procedure in purification steps, the construction of recombinant Lpd was performed. BL21 (DE3) cells carrying the pElpd plasmid were grown at 20 °C for 20 h to maximize the protein yield. Lpd was purified using a HisTrap Chelating column with a recovery of about 99%. His*-*tagged proteins were further purified using a Superose 6 gel filtration column. The enzyme was purified 60-fold with a 51% yield. The molecular weight (58 kD), including a 6xHis-tag of the purified protein was in good agreement with the calculated molecular weight of Lpd. By gel filtration, the active fraction was eluted at a retention time corresponding to approximately 127 kDa (data not shown). The result re-confirmed that Lpd is a dimer in its native state.

### Degradation of each ethoxymer by pure recombinant Lpd

**Figure 4:**
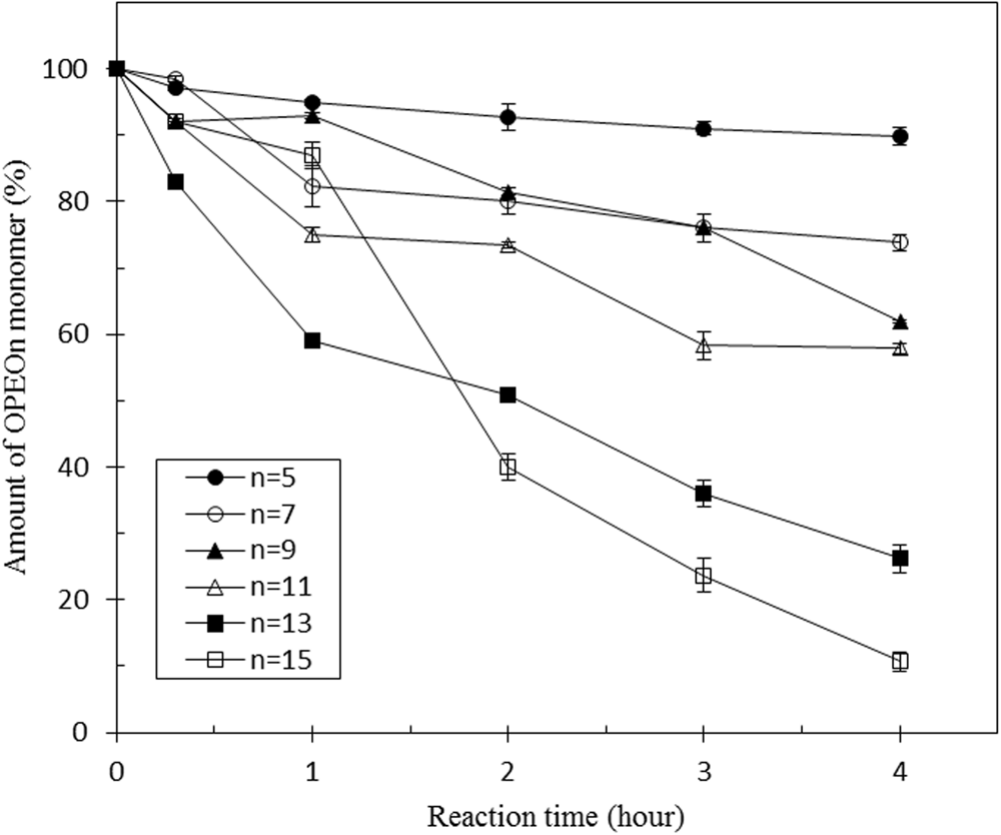
Degradation of OPEO_n_ by purified recombinant Lpd in the presence of metals and NADH. The reaction mixture (4 mL) contained 50 mM KH_2_PO_4_/Na_2_HPO_4_ (pH 8.0), 10 μM MgSO_4_, 5 μM Co(NO_3_)_2_, 100 μM FeSO_4_, 200 μM ZnSO_4_, 10 μM CuSO_4_, 60 μM MnSO_4_, 40 μM Na_2_EDTA, 0.05% OPEO_n_, 96 µg/mL pure Lpd enzyme and 0.5 mM NADH at 30 °C. Total ion count of representative OPEO_n_ (n = 5, 7, 9, 11, 13, 15) is expressed as percentage of the initial ion count of the individual ethoxymer according to the function of reaction time. The ethoxymer containing odd numbers of ethoxylate units represents the degradation of each monomer in this figure. Data points and error bars represent the means and standard deviations of triplicate measurements.

The extent of removal for residual OPEOs during the degradation process by pure recombinant Lpd was also investigated. Figure 4 shows the disappearance of each ethoxymer of the major distribution. The amount of OPEO_n_ decreases as the enzyme-substrate reaction time increases. After 4 hours, the treatment of purified Lpd reduces OPEO_15_ level by approximately 90%. In this figure, we only use the ethoxymers with odd numbers of the ethoxylate units to represent the results. The disappearance rates for OPEO_n_ homologs were OPEO_15_ > OPEO_13_ > OPEO_11_ > OPEO_9_ > OPEO_7_ > OPEO_5_, meaning the longer the ethoxylate chain, the higher chance they were attacked by the reaction generated by this enzyme.

## Discussion

In this study, we investigated the enzyme activities involved in OPEO_n_ degradation in strain TX1. Since the degradation of OPEO_n_ required O_2_ consumption, the oxygen-consuming enzymes were monitored and purified from strain TX1. The pure enzyme was identified as dihydrolipoamide dehydrogenase by its mass and amino acid sequences. The absorption spectrum of the purified Lpd was also studied and showed maximal absorbance at 375 and 458 nm, indicating the presence of FAD as a cofactor in Lpd. The results were consistent with the previous studies, in which Lpd is a homo-dimer^25,28^. Efforts to inactivate the *lpd* gene in strain TX1 was made, however no mutant was obtained. The reason might be due to Lpd serving as a component of some important complex enzyme systems such as the pyruvate dehydrogenase complex and the branched chain ketoacid dehydrogenase complex^29^.

**Figure 5:**
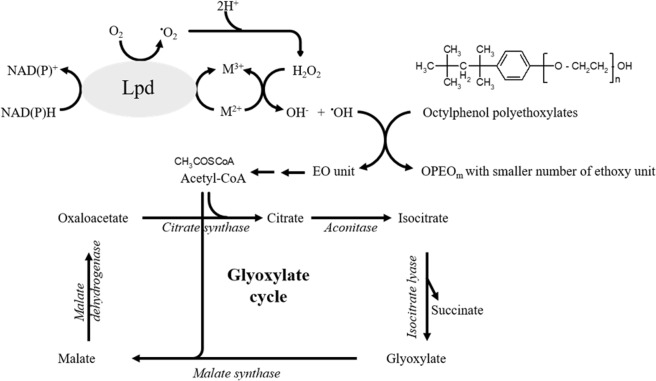
The proposed mechanism for the shortening of the ethoxylated chain of OPEOn involves the attack by OH^•^ radicals that are formed in the presence of lipoamide dehydrogenase, NADH and an excess of metals. The low molecular mass EO units might convert into acetyl-CoA that goes into the glyoxylate cycle to serve as carbon source. M, metals; EO, ethoxylate.

The function of Lpd from TX1 was demonstrated *in vitro* for the degradation of ethoxylate chain containing nonionic surfactants such as Triton X-100. Based on the analysis of OPEO_n_ degraded by Lpd in the presence of metals and an excess of NADH using HPLC-mass spectrometry, the profile of the OPEO_n_ parental ion pattern (the most abundant component is OPEO_9_ (m/z = 625.3)) shifts to lower EO chain products with OPEO_6_ (m/z = 493.3) as the most abundant component after 2 hours reaction. The products detected in this study indicate that the biodegradation mechanism for OPEO_n_ by Lpd involves the cleavage of ethoxylate units in polyethoxylate chains of the surfactant structure. For the shortening of ethoxylate units in polyethoxylate chains during OPEO_n_ degradation, three models – nonoxidative biodegradation, oxidative biodegradation and a mechanism involving the attack by ^•^OH radicals – have been proposed previously^30,31^. Production of hydrogen peroxide and superoxide radical catalyzed by lipoamide dehydrogenase was first reported in 1955 and then confirmed in 1969^32–34^. In addition, when Lpd oxidizes NADH, it also has the ability to reduce ferrous ion presence in the reaction mixture and occurs in a linear proportion manner^35^. The mixture of ferrous ions and H_2_O_2_, namely Fenton’s reagents, will generate hydroxyl radicals according to the reaction: Fe^2+^ + H_2_O_2_ → Fe^3+^ + ^−^OH + ^•^OH^36–40^. The formation of H_2_O_2_ from purified Lpd (TX1) in the presence of metal and an excess of NAD(P)H was investigated in this study. To test the possibility that H_2_O_2_ derived from the Lpd catalyzes the reaction, we measured the production of H_2_O_2_ by using the Amplex Red fluorescent dye. H_2_O_2_ generation was dependent on the presence of Lpd and NADH (Fig. S5). The effect of Lpd concentration on hydrogen peroxide formation was measured by increasing the amount of purified Lpd (0~14 µg). A linear relation between H_2_O_2_ and Lpd was obtained (Fig. S5A). In addition, the effect of NADH on the production of H_2_O_2_ by Lpd was also analyzed by changing the amount of NADH (0~2 mM). A relation between NADH and the production of H_2_O_2_ was directly proportional (Fig. S5B). In addition, the longer the ethoxylate chain of the ethoxymers, the higher was the degradation rate observed (Fig. 4). Therefore, we propose the mechanism for the shortening of the ethoxylated chain of OPEO_n_ involves the attack by ^•^OH radicals that are formed through the Lpd reaction (Fig. 5). In this reaction, Lpd might perform three functions: (1) the oxidation of NAD(P)H^35,41^; the consumption of O_2_ to subsequently generate H_2_O_2_ ^32–34^; and the reduction of metals^35,41^. With the combination of the three reactions (Lpd: NAD(P)H + M^2+^ + O_2_ + H^+^ → NAD(P)^+^ + M^3+^ + ^•^OH + OH^−^), ^•^OH is produced and then attacks the surfactant. The attack by ^•^OH radicals to OPEO_n_ was studied previously^31^. According to Brand *et al*. (1998), three different sites of OPEO_n_ are proposed to be attacked by ^•^OH radicals: (1) CH_2_ and CH_3_ groups of the alkyl chain, (2) the aromatic ring, and (3) CH_2_ groups of the ethoxylate chain. The attack on the CH_2_ groups of the ethoxy chain is highly favored. The attack of the ^•^OH radicals on OPEO_n_ leads to the formation of OPEO_n_ with a smaller number of ethoxy units and low molecular mass units. These low molecular mass units might convert into acetyl-CoA that goes into the glyoxylate cycle (Fig. 5). Thus, this study demonstrates the bacteria can respond to environmental stress by using existing housekeeping enzymes. It might also be applied to the bacterial degradation of other widely used ethoxylated compounds such as polyethylene glycol, Tween and ethoxylated glycerol types of nonionic surfactants.

## Conclusions

Microbes in the environments play a major role in the degradation of widely used surfactants. In this study, *Pseudomonas nitroreducens* TX1 is of special interest because of its capability to use a wide range (0.05–20%) of nonionic ethoxylated surfactants such as octylphenol polyethoxylate (OPEO_n_) as a sole carbon source. We demonstrate a novel catalytic reaction carried out by dihydrolipoamide dehydrogenase involved in the radical formation to attack ethoxylated surfactants which serve as a sole carbon source. It also suggests that a diverse and novel strategy by which bacteria can catabolize a sole carbon source present in the environments.

## Supplementary information

Supplemental Figures
