# Supplementary material for: Bio-Fenton reaction involved in the cleavage of the ethoxylate chain of nonionic surfactants by dihydrolipoamide dehydrogenase from Pseudomonas nitroreducens TX1
Source: Sci Rep. 2019 May 2;9:6827. doi: 10.1038/s41598-019-43266-8 (PMC6497679; doi:10.1038/s41598-019-43266-8)
Supplement: Supplementary file 1 — Supplemental Figures [file 41598_2019_43266_MOESM1_ESM.pdf]

Bio-Fenton reaction involved in the cleavage of the ethoxylate chain of nonionic surfactants by dihydrolipoamide dehydrogenase from *Pseudomonas nitroreducens* TX1

Guo-Chan Hung[a], Ngoc Tuan Nguyen[b], Yu-Ling Sun[a] and Shir-Ly Huang[b, c*]

Department of Life Sciences, National Central University, Jhongli, Taiwan[a]; Institute of Microbiology and Immunology, National Yang-Ming University, Taipei, Taiwan[b]; Institute of Environmental Engineering, National Central University[c]

*Corresponding author:

Shir-Ly Huang, Institute of Microbiology and Immunology, National Yang-Ming University, No. 155, Sec. 2, Li-Nong Street, Taipei 112, Taiwan. Tel.: +886-2-28267108; e-mail: sl.huang@ym.edu.tw.

(Current address for Ngoc Tuan Nguyen: Faculty of Applied Sciences, Ton Duc Thang University, Ho Chi Minh City, Vietnam)

1 M**SQKFDVVVI GAGPGGYVAA IR**AAQLGLKT ACIEKYIGKE GKVALGGTCL
51 NVGCIPSK**AL LDSSWK**YK**EA KEGFEIHGIS TGGVKMDVPA MVAR**K**ANIVK**
101 **NLTGGIATLF KANGVTSFEG HGK**VLANK**QV EVTGLDGK**TQ VLEADNIIIA
151 SGSRPVEIPP APLTEDVIVD STGALEFQSV PK**KLGVIGAG VIGLELGSVW**
201 **ARLGAEVTVL EALDKFLPAA DEQIAKEALK TLTK**QGLNIR LGARVTGSEV
251 KKKQVTVAFT DANGEQKETF DKLIVAVGR**R PVTTDLLAAD SGVTLDER**GF
301 IYVDDHCKTS VPGVYAIGDV VR**GAMLAHKA SEEGVMVAER** IAGHKAQMNY
351 DLIPSVIYTH PEIAWVGK**TE QQLKGEGVEV NVGTFPFAAS GRAMAANDTG**
401 **GLVKVIADAK TDR**VLGVHVI GPSAAELVQQ GAIGMEFGTS AEDLGMMVFS
451 HPTLSEALHE AALAVNGHAI HIANRKKR

Supplemental Figure S1. LC-MS/MS identification of the purified protein. After LC-MS/MS analysis, 52-kDa protein band peptide sequence matches to dihydrolipoamide dehydrogenase from *P. nitroreducens* TX1 (accession number WP_017518066) are shown in bold.

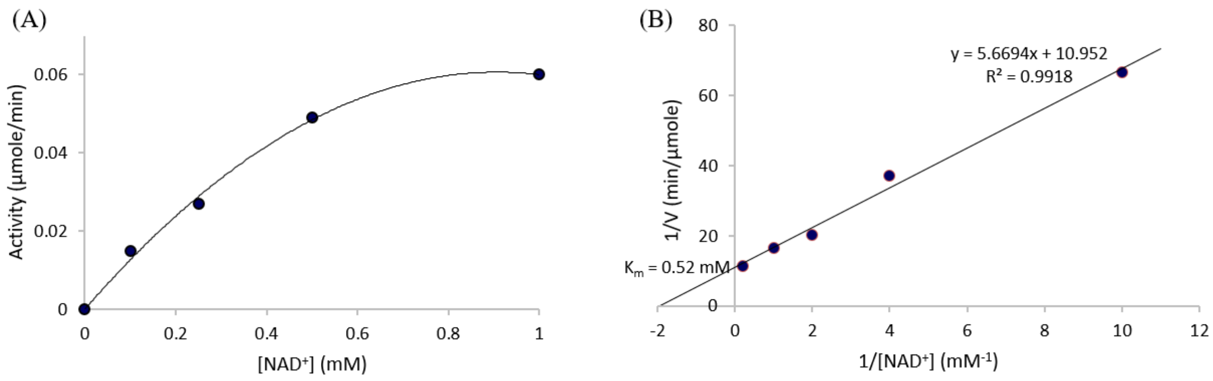

Supplemental Figure S2. Enzyme kinetics of purified Lpd from *P. nitroreducens* TX1 using NAD^+ as co-substrate. (A) Plot of NAD^+ concentration versus enzyme activity. (B) Lineweaver-Burk of NAD^+ concentration versus enzyme activity to demonstrate the K_m value. The reaction rate is recorded as absorbance at 340 nm, 30°C, in 50 mM KH_2PO_4/Na_2HPO_4 (pH 8.0).

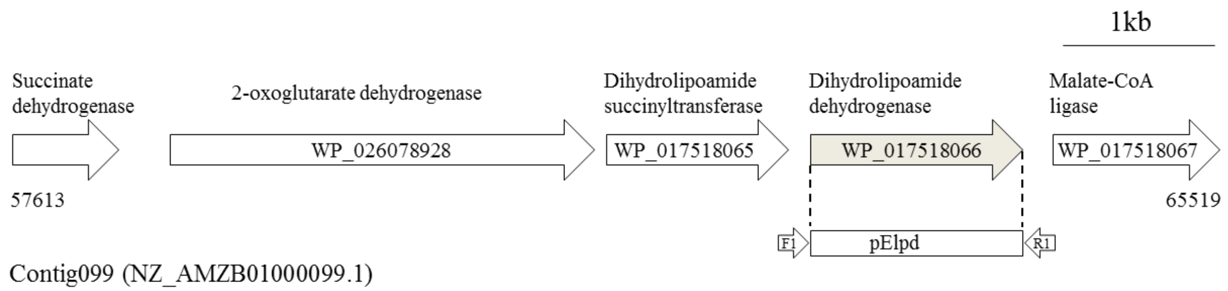

Supplemental Figure S3. Genetic organization of 7.9 kb fragment (location 57613-65519) containing dihydrolipoamide dehydrogenase gene from contig099 in *P. nitroreducens* TX1. Arrows indicate the orientation of each gene. The corresponding accession number for each protein is shown.

```
ATGAGCCAGAAATTCGACGTGGTTGTGATTGGTGCCGGCCCCGGCGGCTACGTAGCCGCCATCC
GTGCCGCCCAACTCGGCCTGAAGACCGCTTGCATCGAGAAGTACATCGGTAAAGAGGGCAAGGT
CGCTCTCGGCGGTACCTGCCTGAACGTAGGCTGCATTCCGTCCAAGGCGCTGCTGGACAGCTCC
TGGAAGTACAAGGAAGCCAAAGAAGGTTTCGAGATTCACGGTATCTCCACCGGCGGCGTGAAGA
TGGACGTCCCGGCGATGGTTGCCCGCAAGGCCAACATCGTGAAGAACCTGACCGGCGGCATCGC
TACCCTGTTCAAGGCCAACGGCGTGACTTCCTTCGAAGGCCACGGCAAGGTCCTGGCCAACAAG
CAGGTCGAAGTGACCGGCCTGGACGGCAAGACCCAGGTGTTGGAAGCCGACAACATCATCATCG
CCTCGGGCTCCCGTCCGGTGGAAATCCCGCCGGCTCCGCTGACCGAAGACGTGATTGTCGATTC
CACCGGCGCCCTGGAATTCCAGAGCGTACCCAAGAAGCTGGGCGTGATCGGTGCTGGCGTTATC
GGCCTGGAACTGGGTTCGGTCTGGGCTCGCCTGGGCGCTGAAGTCACCGTCCTGGAAGCCCTGG
ACAAGTTCCTCCCGGCTGCCGACGAGCAGATCGCCAAGGAAGCGCTGAAGACCCTGACCAAGCA
AGGCCTGAACATCCGCCTGGGCGCTCGCGTCACCGGTTCGGAAGTGAAGAAGAAGCAGGTCACC
GTGGCCTTCACCGATGCCAACGGCGAGCAGAAGGAAACCTTCGACAAGCTGATCGTGGCCGTGG
GCCGTCGGCCGGTGACCACCGATCTGCTGGCTGCGGACAGCGGCGTGACCCTGGACGAGCGCGG
TTTCATCTACGTCGACGACCACTGCAAGACCAGCGTTCCGGGCGTCTACGCCATCGGTGATGTG
GTCCGTGGCGCCATGCTGGCGCACAAGGCCTCGGAAGAGGGCGTGATGGTTGCCGAGCGCATCG
CCGGCCACAAGGCCCAGATGAACTACGACCTGATTCCGTCGGTGATCTACACCCACCCGGAAAT
CGCATGGGTCGGCAAGACCGAGCAGCAGCTCAAGGGCGAAGGCGTCGAAGTCAACGTCGGCACC
TTCCCGTTCGCCGCCAGCGGCCGCGCCATGGCTGCCAACGACACCGGCGGCCTGGTCAAGGTCA
TCGCCGATGCCAAGACCGACCGCGTACTGGGCGTCCACGTGATCGGCCCGAGCGCCGCCGAGCT
GGTTCAGCAGGGCGCGATCGGCATGGAATTCGGCACCAGTGCCGAAGACCTGGGCATGATGGTC
TTCTCCCACCCGACTCTGTCCGAAGCGCTGCACGAAGCGGCACTGGCAGTGAATGGCCACGCCA
TCCACATCGCCAACCGCAAGAAGCGCTAA
```

Supplemental Figure S4. DNA sequence of dihydrolipoyl dehydrogenase gene from *Pseudomonas nitroreducens* strain TX1

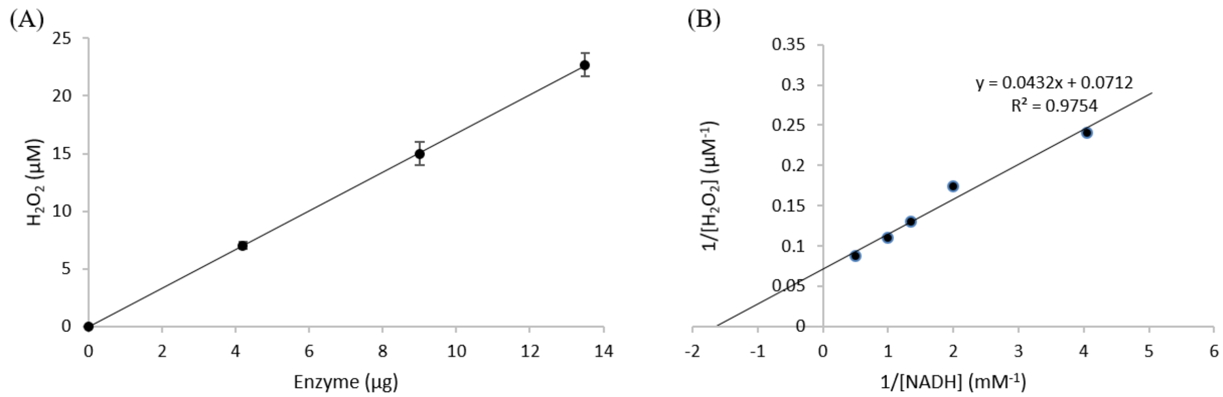

Supplemental Figure S5. Hydrogen peroxide production by purified Lpd from *P. nitroreducens* TX1. (A) Plot of Lpd concentration versus H_2O_2 production. (B) Lineweaver-Burk of NADH concentration versus H_2O_2 production. 0.25 mL reaction mixture containing 50 mM KH_2PO_4/Na_2HPO_4 (pH 8.0), 0~2 mM NADH, 0.2 mM $ZnCl_2$ and 0~14 µg pure Lpd at 30°C for 1 h and then horseradish peroxidase (0.2 U/ml) and amplex red reagent (1 µM) is added. Production of resorufin is followed by an increasing absorbance at 571 nm.
